# High Prevalence of Low-Level Parasitemia With *Plasmodium vivax* in Makira-Ulawa Province Presents a Challenge for the Diagnosis and Eradication of Malaria in Solomon Islands

**DOI:** 10.31486/toj.20.0023

**Published:** 2021

**Authors:** James Fink, Peter D. Jones

**Affiliations:** Department of Health Sciences and Medicine, Bond University, Robina, Gold Coast, Queensland, Australia

**Keywords:** *Artemisinins*, *malaria*, *malaria–falciparum*, *malaria–vivax*, *Melanesia*, *point-of-care testing*, *primaquine*

## Abstract

**Background:** Malaria remains endemic in Solomon Islands, but data on malaria in the provinces of Solomon Islands are limited. This study from Makira-Ulawa Province aimed to identify the most prevalent strain of malaria and assess if the available rapid diagnostic test (RDT) was effective in Kirakira Hospital.

**Methods:** Forty-five patients who presented to Kirakira Hospital with symptoms of fever had a positive malaria parasite smear during a 4-week period in 2017. The parasite count for each smear was calculated. Simultaneous testing using the CareStart Malaria HRP2/pLDH (Pf/pan) Combo RDT was conducted. The data for all malaria parasite smears performed in Makira-Ulawa Province in 2016 were collated for comparison.

**Results:** All 45 patients diagnosed with malaria in a 4-week period in 2017 were positive for *Plasmodium vivax*. The median parasite load was 280 parasites per μL (range, 160 to 640 parasites per μL). None of the 45 CareStart RDTs performed was positive. In 2016, 5,505 of 17,195 patients (32.0%) screened had malaria parasites detected on a malaria parasite smear. *P vivax* was detected in 5,212 (94.7%) and *Plasmodium falciparum* in 285 (5.2%) of patients with malaria.

**Conclusion:** *P vivax* is the predominant strain of malaria present in Makira-Ulawa Province. RDTs were not helpful in the diagnosis of malaria at Kirakira Hospital. The parasite load detected in the 45 patients diagnosed with malaria in this study was low. A focus on attempting to eradicate *P vivax* in the community through improved compliance with treatment protocols is suggested as a possible way forward to best manage malaria in Makira-Ulawa Province.

## INTRODUCTION

Malaria remains endemic in Solomon Islands, but progress toward eradication has been slow and challenging. Despite a reported reduction of 89% in confirmed malaria cases from 1993 to 2011,^1^ wide discrepancies exist in the reported incidences across the 9 provinces of Solomon Islands. In 2011, rates ranged from 1.6 cases per 1,000 population in Isabel Province to 96.8 cases per 1,000 population in Guadalcanal Province.^2^

Since 2009, the diagnosis and treatment of malaria in Solomon Islands has been available free of charge through public health facilities. Artemisinin-based combination therapy (ACT) has been the first-line treatment for all febrile cases of acute malaria, with 14 days of primaquine used for radical treatment of *Plasmodium vivax*. Glucose-6-phosphate dehydrogenase (G6PD) screening has been a requirement prior to commencing primaquine treatment.^3^

Limited data are available about malaria in Makira-Ulawa Province of Solomon Islands. Makira-Ulawa is the fifth largest province by population, estimated at 52,000 inhabitants in 2017, and is located approximately 180 km southeast of Honiara, the capital city of Solomon Islands.^4^ Makira-Ulawa has one small hospital located in the provincial capital Kirakira. Trained microscopists perform as many as 50 malaria parasite smears each day at Kirakira Hospital. The hospital also has a store of rapid diagnostic test (RDT) cartridges that have been shown to be accurate in diagnosing acute malaria cases in other settings. However, the nurses at Kirakira Hospital had stopped using the RDT cartridges because they were “never positive” and “did not work.” No specific data were available on when the nurses stopped using the RDTs, but they had not been observed using them since Bond University medical students started completing placements in Kirakira Hospital in 2013.

In response to these practical observations, we performed a small prospective study of patients presenting to Kirakira Hospital with fever and a positive malaria parasite smear during a 4-week period in 2017. Each of these patients also underwent an RDT cartridge test. In addition, we reviewed the laboratory results for all malaria parasite smears performed during 2016 in Makira-Ulawa Province.

### Information About Malaria

Malaria is a mosquito-borne infectious disease that, according to the World Health Organization (WHO), is responsible for more than 200 million episodes of illness per year across the globe, resulting in up to 400,000 deaths annually. Malaria is caused by the transmission of a parasite from the female *Anopheles* mosquito into humans. The disease has 4 known strains, but 2 strains—*Plasmodium falciparum* and *P vivax*—are responsible for the majority of clinical disease globally. Malaria disproportionately affects the poorest countries of the world, with 85% of the total disease burden borne by Africa and India.^5^ United Nations Sustainable Development Goal 3.3 is to end the epidemics of acquired immunodeficiency syndrome, tuberculosis, and malaria by 2030.^6,7^ Attaining this goal for malaria is looking increasingly out of reach for the Pacific countries.

*P falciparum* is the primary strain responsible for severe malaria in Africa and India and accounts for more than 90% of cases of malaria globally. *P vivax* is principally seen in Southeast Asia, South America, and the Western Pacific. Although the acute illness associated with *P vivax* malaria is less severe than the illness associated with *P falciparum, P vivax* has the potential to cause recurrent infection because it can lie dormant in the liver. Chronic infection with *P vivax* malaria can cause anemia and splenomegaly, can adversely affect pregnancy, and is a significant cause of morbidity and mortality in Solomon Islands.

Since 2001, the WHO has recommended the use of ACT for acute treatment of malaria because of its efficacy against both the *P falciparum* and *P vivax* strains.^8^ Since the introduction of ACT, the number of malaria cases has been reduced in the Southwestern Pacific. ACT was made the first-line treatment for acute malaria in Solomon Islands in 2009, and during the next 6 years to 2015, the number of malaria cases in Solomon Islands dropped by 75%.

From 2015 to 2020, however, the rate of decline in the number of malaria cases slowed in Solomon Islands. The 2019 malaria report from the WHO identifies *P falciparum* as responsible for more than 60% of malaria in Solomon Islands.^5^ However, evidence suggests that *P vivax* is now the overwhelming predominate strain of malaria in the provinces.^9^ Actual data from the remote provinces of Solomon Islands are very limited; the WHO figures are calculated estimates. Research from Kirakira has shown that these estimates can be very different from actual clinical data taken from the medical records in Solomon Islands.^10^

## METHODS

Ethics approval to conduct the research was granted by the Human Research Ethics Committee of Bond University (BURO 15397).

Patients presenting to the outpatient department of Kirakira Hospital with a fever during April 2017 had a malaria parasite smear taken and assessed by microscopists who identified the strain of malaria and recorded the number of parasites per 200 white blood cells. The number of parasites per μL is estimated by assuming an average count of 8,000 white blood cells per μL, so 1 parasite per 200 white blood cells is equivalent to 40 parasites per μL. A count of <1,000 parasites per μL is considered a low level of parasitemia in acute malaria.

Consent was obtained from patients with a positive malaria parasite smear to have an RDT performed on their blood sample.^11,12^ RDTs were only performed on patients with a positive malaria smear.

The available RDT at Kirakira Hospital was the CareStart Malaria HRP2/pLDH (Pf/pan) Combo. The CareStart RDT detects the histidine-rich protein 2 antigen of *P falciparum* and *Plasmodium* lactate dehydrogenase that is produced by all 4 species of malaria.^13^

The microscopists at Kirakira Hospital evaluate malaria parasite smears for the entire province. Outside of Kirakira Hospital, nurses at nurse-run health clinics assess patients for fever and send malaria parasite smears to the hospital. We audited all of the malaria parasite smears evaluated at Kirakira Hospital in 2016. The only information available from the audit was the strain of malaria. No information about the parasite load could be retrieved.

## RESULTS

In April 2017, 45 patients who presented with a fever to Kirakira Hospital had a positive malaria parasite smear. All patients were found to have *P vivax*. The median parasite load was 7 per 200 white blood cells or 280 parasites per μL. The highest parasite load detected was 16 per 200 white blood cells or 640 parasites per μL (Table 1). In contrast, none of the 45 CareStart RDTs performed was positive. No patient had a clinically severe case of malaria. All patients were managed as outpatients, and all patients recovered.

**Table 1.** Malaria Parasite Smear (MPS) Results for Febrile Patients Presenting to Kirakira Hospital in April 2017

MPS Positive	Malaria Species	Parasites Observed per 200 White Blood Cells	Calculated Parasite Load
45	*Plasmodium vivax*	Median: 7/200 Range: 4-16/200	Median: 280/μL Range: 160-640/μL

Note: One parasite per 200 white blood cells is calculated as being the equivalent of 40 parasites per μL.

In 2016, 17,195 malaria parasite smears were evaluated at Kirakira Hospital, resulting in 5,212 positives for *P vivax*, 285 positives for *P falciparum*, and 8 mixed species positives (Table 2). These results show that in 2016, 32.0% of febrile patients seen in outpatient settings in Makira-Ulawa Province had positive malaria parasite smears and that *P vivax* was detected in 94.7% of cases, demonstrating that *P falciparum* is an uncommon strain of malaria detected in Makira-Ulawa Province.

**Table 2.** Malaria Parasite Smear (MPS) Results From the Kirakira Hospital Malaria Laboratory in 2016

Test/Results	Adults	Infants	Total	Percentage
MPS Performed	16,291	904	17,195	N/A
MPS Positive	5,284	221	5,505	32.0^a^
*Plasmodium vivax*	5,003	209	5,212	94.7^b^
*Plasmodium falciparum*	273	12	285	5.2^b^
Mixed species	8	0	8	0.1^b^

^a^Percentage of positive MPS tests overall (5,505/17,195).

^b^Percentage of positive MPS tests.

## DISCUSSION

Our study confirmed the suspicion of the local nursing staff that the RDTs were not effective for diagnosing malaria in Kirakira. This result is at odds with the reported sensitivities of RDTs that range between 75% and 90%. However, the studies of RDT sensitivities have principally been conducted in the sub-Saharan region of Africa where *P falciparum* is the main species, the average parasite loads are usually much greater than 1,000 parasites per μL, and levels can be as high as 30,000 parasites per μL.^14^ The WHO recommends the use of quality RDTs as standard diagnostic tools for routine malaria case management, but evidence is insufficient to assess if RDTs are useful in low-transmission settings where submicroscopic infections, often not associated with febrile illness, have been shown to have efficient gametocyte production that serves as an ongoing reservoir of potential transmission.^15^ Further research into the sensitivity of the RDTs confirms that a parasite load of >500 parasites per μL is often required to trigger a positive result, so RDTs are frequently unable to detect malaria in patients with low parasite loads. In our study, 4 patients had parasite loads >500 parasites per μL, but all had negative RDT results.

The failure of the RDTs in this study could have several explanations. The kits may have been defective, the staff may not have used them correctly, and the low parasite loads may have contributed to the ineffectiveness of the RDTs. Because we only performed RDTs on patients with positive malaria parasite smears, we could not confirm a false-positive rate for this particular RDT that has been noted by Swiss researchers conducting fieldwork in Eswatini, formerly known as Swaziland.^16^ RDTs are an important tool for the detection of malaria in resource-poor communities without access to microscopy; however, we had no success with the RDT that was available. Kirakira Hospital had no access to other RDTs that can distinguish between *P falciparum* and *P vivax* strains and potentially might have been more effective than the CareStart Malaria HRP2/pLDH (Pf/pan) Combo RDT.^17^ The total budget for all health care in Makira-Ulawa Province is only $1.3 million US dollars per annum or the equivalent of $26 US dollars per annum per capita, so health care resources are extremely limited.^18^

Our study results demonstrate what appears to be a dramatic change in the epidemiology of malaria in Makira-Ulawa Province. *P falciparum* in this audit was rare and accounted for only 5.2% of identified cases vs the WHO estimate of approximately 60%.^5^ A possible reason for this change is the introduction of ACT protocols that are highly effective against *P falciparum* and have been first-line treatment for all cases of malaria in Solomon Islands since 2009. The staff in the Kirakira Hospital malaria laboratory provided anecdotal evidence that the clinics where *P falciparum* was detected were remote and did not have access to ACT. The introduction of ACT in 2009 proved hugely successful in eradicating *P falciparum* in Solomon Islands. In Makira-Ulawa Province, however, the situation with *P vivax* is quite different.

*P vivax* malaria requires treatment with primaquine for 2 weeks to prevent recurrent infection. However, primaquine can cause acute hemolytic anemia in patients with G6PD deficiency. In Solomon Islands, research indicates that approximately 7% of the population has a deficiency of G6PD and would be at risk of developing a severe hemolytic reaction when exposed to primaquine.^19^ Consequently, since 2009, the Ministry of Health for Solomon Islands has appropriately recommended that all patients with *P vivax* malaria be screened for G6PD deficiency, and if safe, to proceed with primaquine treatment.^20^

Implementing this treatment protocol across Makira-Ulawa Province has been challenging on many levels. First, health literacy in the community is low, and the need for primaquine treatment when the patient has recovered from the acute febrile episode is often poorly understood by the nursing staff and the patients. Second, adherence with outpatient medication is a particularly challenging problem in Solomon Islands. One strategy to overcome lack of adherence would be to directly observe patients taking their primaquine medication each day to ensure compliance, but resources to implement such a policy are limited. Third, the G6PD screening test is not available at all health centers in Makira-Ulawa Province, and the community and health workforce do not have a clear understanding of why the test is important.^21^

An important issue raised by the study data is how the diagnosis of acute malaria is made. The presence of a fever and a positive malaria parasite smear have been considered sufficient to establish a diagnosis of malaria. What we cannot extract from this audit is whether the low-level parasitemia was the cause of the fever or if patients had other common causes of fever such as acute viral infection. The patients diagnosed with malaria in this study were not sick enough to require inpatient treatment and were effectively managed on an outpatient basis. The highest parasite load among the 45 patients in this study was 640 parasites per μL (16 parasites per 200 white blood cells). This number is below the level likely to cause symptoms of severe malaria (defined as >1,000 parasites per μL or >25 parasites per 200 white blood cells) and explains why all patients could be successfully managed as outpatients.

Whether the low-level parasitemia identified in this study was sign of a chronic underlying illness and not the cause of the fever is a vexing question because no agreed-upon levels of parasitemia that indicate a definite diagnosis have been established.^22^ Further, what constitutes symptomatic vs asymptomatic malaria has not been firmly established. Chronic or recurrent malaria has long been recognized to cause long-term health problems such as chronic anemia and splenomegaly.^23^

To answer the question of whether the low-level parasitemia identified in this study is a sign of a chronic underlying illness and not the cause of the fever would require screening the population to determine the prevalence of low-grade parasitemia, anemia, and clinical splenomegaly in asymptomatic patients. Such population screening is not part of the current national strategy to address malaria in Solomon Islands.^11^ The number of cases of malaria detected in the province would have likely been higher if polymerase chain reaction (PCR) had been used to screen for malaria because this technology is more sensitive in identifying malaria than microscopy or RDT.^24^ Asymptomatic malaria is being recognized with increasing frequency in Southeast Asia, with research using PCR technology revealing that 3 to 4 times as many patients with malaria may be detected with PCR screening alone.

Developing an implementable combination of diagnostic testing and treatment to try to eradicate malaria from Makira-Ulawa Province will be an ongoing challenge.^25^ Malaria is a complex infection; the challenge is to understand the nature of the illness and how that nature might change as the epidemiology changes. Therefore, developing a better understanding of low parasite level and asymptomatic disease is necessary for eradication programs to be successful.

## CONCLUSION

The epidemiology of malaria in Makira-Ulawa Province has changed, with *P vivax* representing 94.7% of diagnosed cases and *P falciparum* having been almost eradicated from the province. To meet the goal of Makira-Ulawa being a malaria-free province by 2030, population screening is needed to determine the prevalence of low-level parasitemia in the province and if a significant burden of chronic asymptomatic *P vivax* infection exists. Such a study should be followed by a coordinated public health and implementation campaign that educates the public and health workforce about the importance of adherence to treatment strategies for the eradication of *P vivax* that include screening for G6PD deficiency and a 14-day course of primaquine.

## Notes

This article meets the Accreditation Council for Graduate Medical Education and the American Board of Medical Specialties Maintenance of Certification competencies for Patient Care, Medical Knowledge, and Practice-Based Learning and Improvement.
